# QTL Mapping and Favorable Allele Mining of Nitrogen Deficiency Tolerance Using an Interconnected Breeding Population in Rice

**DOI:** 10.3389/fgene.2021.616428

**Published:** 2021-04-06

**Authors:** Congcong Shen, Kai Chen, Yanru Cui, Jiantao Chen, Xuefei Mi, Shuangbin Zhu, Yajun Zhu, Jauhar Ali, Guoyou Ye, Zhikang Li, Jianlong Xu

**Affiliations:** ^1^Shenzhen Branch, Guangdong Laboratory for Lingnan Modern Agriculture, Agricultural Genomics Institute at Shenzhen, Chinese Academy of Agricultural Sciences, Shenzhen, China; ^2^Institute of Crop Sciences, National Key Facility for Crop Gene Resources and Genetic Improvement, Chinese Academy of Agricultural Sciences, Beijing, China; ^3^College of Agronomy, Hebei Agricultural University, Baoding, China; ^4^International Rice Research Institute, Los Baños, Philippines

**Keywords:** GWAS, interconnected breeding population, nitrogen deficiency tolerance, favorable allele mining, pyramiding breeding

## Abstract

Nitrogen is one of the most important nutrients for rice growth and development. Breeding of nitrogen deficiency tolerance (NDT) variety is considered to be the most economic measure to solve the constrain of low nitrogen stress on grain yield in rice. An interconnected breeding (IB) population of 497 lines developed using Huanghuazhan (HHZ) as the recurrent parent and eight elite lines as the donor parents were tested for five traits including grain yield, biomass, harvest index, thousand grain weight, and spikelet fertility under two nitrogen treatments in three growing seasons. Association analysis using 7,388 bins generated by sequencing identified a total of 14, 14, and 12 QTLs for the five traits under low nitrogen (LN), normal nitrogen (NN), and LN/NN conditions, respectively, across three seasons. Favorable alleles were dissected for the 40 QTLs at the 10 NDT regions, and OM1723 was considered as the most important parent with the highest frequency of favorable alleles contributing to NDT-related traits. Six superior lines all showed significantly higher GY in LN environments and similar GY under NN environments except for H10. Substitution mapping using near-isogenic introgression lines delimited the *qTGW2-1*, which was identified on chromosome 2 under LN, NN, and LN/NN conditions into two QTLs, which were located in the two regions of about 200 and 350 kb with different favorable alleles. The bins 16, 1301, 1465, 1486, 3464, and 6249 harbored the QTLs for NDT detected in this study, and the QTLs/genes previously identified for NDT or nitrogen use efficiency (NUE) could be used for enhancing NDT and NUE by marker-assisted selection (MAS).

## Introduction

Rice (*Oryza sativa* L.) is one of the most important staple crops in the world and also the main calorie source for more than 65% of the population in China. Nitrogen is one of the most important nutrients in the growth stage of crops (Yoshida, 1981). In China, about 70% of the rice paddy fields have low or moderate nitrogen supply, which limits yield potential of rice production (Feng et al., 2018). Therefore, development of rice varieties with good nitrogen deficiency tolerance (NDT) is considered as a key method of sustainable agriculture for food security.

In recent years, many QTLs/genes for NDT, measured as the relative trait values under low nitrogen (LN) stress to normal nitrogen (NN) conditions have been reported by QTL mapping and gene expression methods. QTLs were detected for the traits or the relative trait values of seedling height, shoot dry weight, chlorophyll content (Tong et al., 2011), maximum root length, root dry weight, plant dry weight (Lv et al., 2010; Zhao et al., 2014) at seedling stage, spikelet fertility percentage (Shan et al., 2005), grain yield (Tong et al., 2006), panicle number and total panicle weight (Wang et al., 2009), grain number per panicle, spikelet fertility percentage and 100-grain weight (Tong et al., 2011), and nitrogen efficiency (Tang et al., 2011) at maturity stage using populations such as backcross, chromosome segment substitution line, and recombinant inbred lines under LN stress or different nitrogen level conditions. A total of 14 QTLs for NDT-related traits (relative shoot and root biomass, and relative plant height) were identified by Lian et al. (2005) in an RIL population derived from Zhenshan97 × Minghui63. Fifteen QTLs were identified for the four NDT traits including relative grain yield, relative biomass, relative grain nitrogen, and relative biomass nitrogen (Wei et al., 2012), and some of which are close to genes controlling nitrogen cycle.

Most of NDT QTLs reported in rice were conducted using populations derived from two parents and sparse linkage maps constructed using restriction fragment length polymorphism (RFLP) or simple sequence repeat (SSR). It is very hard to obtain precise information about the QTLs using a small number of markers, which were coarsely located. Fine mapping using a large secondary population and new markers are needed (Wang et al., 2014). With the development of sequencing technology, high-density single-nucleotide polymorphism (SNP) markers can be easily and quickly generated, which has been widely applied to genome-wide association studies (GWAS) and QTL mapping in rice and many other crops (Yan et al., 2010; Wang et al., 2014; Chen et al., 2020).

The past decade has seen the rise of multiparental populations as a study design offering great advantages for genetic studies in plants. Multiparent mapping populations such as nested association mapping (NAM) population (Yu et al., 2008) and multiparent advanced generation inter-cross (MAGIC) population (Cavanagh et al., 2008) have been developed for many crops. A NAM population is usually generated by crossing multiple genotypes with a single genotype. Using multiparental population for QTL mapping provides an opportunity to test pleiotropy, genetic background effect, and the genetic overlap between different complex traits (Qu et al., 2020). Buckler et al. (2009) developed a NAM population that was consisted of 25 families with 200 lines per family in maize. The NAM population has been used for studying the complex traits in maize, such as pathogen resistances (Kump et al., 2011), morphological traits (Tian et al., 2015), and kernel composition (Cook et al., 2012). Jordan et al. (2011) developed a NAM population containing 56 families for detecting QTLs in sorghum. However, the application of NAM population for QTL mapping in rice is rarely reported.

A backcross (BC)-based breeding strategy has been adopted by our team to improve multiple abiotic stress tolerance for many years (Ali et al., 2006; He et al., 2010; Meng et al., 2013; Wang et al., 2013; Feng et al., 2018). A few outstanding varieties were used as recurrent parents, and many varieties and landraces were used as donor parents in combination with a selection of tolerance to multiple stresses and grain yield. As a result, many small-to-medium size populations sharing a common parent were developed (Ali et al., 2017). Mapping QTL for a range of complex traits using these populations has been carried out (Li et al., 2005; Cui et al., 2015; Feng et al., 2018). However, the interconnectedness between the populations has not been well exploited. A set of breeding populations linked together by a common parent can be regarded as a single interconnected breeding (IB) population for mapping purpose, which is similar to a NAM population that consists of subpopulations sharing a common parent. It is expected that IB population could significantly increase the mapping resolution and power by exploring multiple populations simultaneously. An IB population consisting of highly selected introgression lines derived using HHZ as recurrent parent and eight elite lines as donors has been successfully used in identifying QTL for cold tolerance at the booting stage (Zhu et al., 2015). The fact that a QTL for cold tolerance was fine mapped to a 192-kb region encouraged us to explore this IB population for other traits.

The current study was to identify QTL and favorable alleles for NDT at the reproductive stage. The objectives of this study were (1) to identify the QTLs affecting NDT under different nitrogen conditions; and (2) to gain a better understanding of the genetic relationships between NDT and GY at the QTL level. The results will provide a good example of QTL mapping using breeding population and useful information for rice breeding of NDT by marker-assisted selection (MAS).

## Materials and Methods

### Plant Materials and Field Experiments

The HHZ IB population used in the study was derived from recurrent parent Huanghuazhan (HHZ) crossed with eight donor parents IR50, IR64, Teqing, PSBRc28, PSBRc66, CDR22, OM1723, and Phalguna. Eight F_1_s were backcrossed once with HHZ to harvest the BC_1_F_1_. Through multiple generations selfing and screenings for yield and abiotic stresses (drought, salinity, and submergence), a total of 496 BC_1_F_5_ lines were produced (Ali et al., 2017). A total of 496 lines of the IB population plus the HHZ were field tested under different nitrogen conditions in the early and late seasons of 2013 (2013E and 2013L) and the early season of 2014 (2014E) in Shenzhen (22°52′N 113°46′E), Guangdong province, China. Two nitrogen conditions were applied, they were (1) low nitrogen condition (LN), no chemical nitrogen fertilizer was applied to the LN paddy since 2012; (2) normal nitrogen condition (NN), 150 kg N ha^–1^ was applied with two splits. About 70% nitrogen fertilizers were used as basal, and 30% nitrogen fertilizers were applied at 15 days after transplanting under the normal nitrogen condition. Phosphorus (40 kg ha^–1^) and potassium (40 kg ha^–1^) were applied as basal under two nitrogen conditions. All fertilizers were broadcast by hand. The LN paddy soil had the following properties: pH 6.18, organic matter of 4.32 g kg^–1^, total N of 380 mg kg^–1^, available P of 59.2 mg kg^–1^, and available K of 165 mg kg^–1^. The key properties of the NN paddy soil were pH 5.97, organic matter of 10.9 g kg^–1^, total N of 920 mg kg^–1^, available P of 88.8 mg kg^–1^, and available K of 155 mg kg^–1^.

The experiments were conducted using a randomized block design with two replications. The seeds were sowed on March 5 in 2013E and 2014E, and on July 13 in 2013L. The seedlings were transplanted into three rows with eight plants per row at a planting density of 20 cm × 16.5 cm on April 5 in 2013E and 2014E, and on August 5 in 2013L. The field management was according to the local rice production, and the pests, diseases, birds, weeds, and rats were intensively controlled during the whole growth duration.

### Sampling and Trait Measurement

At maturity stage, six uniform plants from the middle of each plot were harvested. The plants were cut at ground level, and grains were separated from other parts (leaves and sheath, and stems and panicle branches). All samples were oven-dried at 80°C until a constant weight was achieved. Grain yield per plant (GY), biomass per plant (BM), thousand grain weight (TGW), and spikelet fertility (SF) were measured. Harvest index (HI) was calculated by GY/BM. Also, specific parameters were calculated using the following equations:

Relative harvest index (RHI) = HI_LN_/HI_NN_,
Relative grain yield (RGW) = GY_LN_/GY_NN_,
Relative biomass yield (RBM) = BM_LN_/BM_NN_,
Relative thousand grain weight (RTGW) = TGW_LN_/TGW_NN_,
Relative spikelet fertility (RSF) = TSF_LN_/TSF_NN_.

### SNP Genotyping and Bin Markers

To get the consensus sequence that can be used to compare the differential bases between the HHZ and the donor parent, all reads from each parent with 30× re-sequencing were first aligned to the Nipponbare reference genome. Based on the differential SNP results, the pseudo-molecules of both parents were generated in pairs by using Perl script for eight mapping populations, respectively. A total of 400 k high-quality SNPs was developed based on whole-genome re-sequencing of 2× in IB population. To improve analytical accuracy and the resolution of re-sequencing data, we used the sliding window approach to determine the recombination breakpoint based on the SNP ratios (Huang et al., 2009). Based on the recombination breakpoint, the 496 lines were aligned to all the chromosomes and compared for the minimal of 50-kb intervals. Adjacent 50-kb intervals with the same genotype across the entire IB population were recognized as a single recombination bin. A total of 7,388 bins covered in all of 12 chromosomes were obtained for association analysis.

### Statistical and Association Analyses

We used the R project for statistical analysis. The basic statistics of phenotypic values were calculated using the R package named EnvStats. Correlation coefficients were obtained from the R function called corr. Two-way ANOVA was conducted by the R function called ANOVA. Based on the adjusted means and the trait ratios of LN to NN of ILs, association analyses were conducted by R-package MAGICqtl^1^. This package implemented random model-based methods proposed by Wei and Xu (2016) for QTL detection using a multi-parental population. The initial threshold to declare a significant association was set at *p* = 1.0 × 10^–4^. The threshold value of the main effect QTL was LOD > 3.2. The gene effects of multiple alleles in each QTL were estimated by the difference between the mean phenotypic value of one allele and mean phenotypic value of all the alleles.

## Results

### Phenotypic Performance

Grain yield and BM were much higher in the NN than in the LN, while HI and SF were similar in the two nitrogen treatments (Table 1). TGW was slightly higher in the NN. The average GY, BM, HI, SF, and TGW of the IB population were similar to those of HHZ. The estimates of skewness ranged from −2.873 to 1.783, and the estimates of kurtosis ranged from 0.059 to 16.65. The heritability in the NN was similar to or higher than that in the LN in all three seasons, ranging from 0.647 of BM under LN in 2013E to 0.929 of HI under NN in 2014E.

**TABLE 1:** Performances of HHZ and IB population under LN and NN conditions in three seasons.

HHZ				IB population					
Trait^a^		Season^b^	Mean	Mean	Min	Max	Skew	Kurt	*H_*B*_^2^*
GY	LN	2013E	10.0	10.3	3.98	26.61	0.775	1.528	0.650
		2013L	6.6	6.2	1.93	16.13	0.753	2.074	0.718
		2014E	10.1	9.5	2.47	27.16	1.442	4.527	0.740
	NN	2013E	25.1	21.6	6.98	35.50	0.032	0.059	0.747
		2013L	17.3	17.7	4.12	34.25	0.380	0.701	0.685
		2014E	22.0	20.0	7.77	46.68	1.046	2.326	0.745
BM	LN	2013E	16.3	19.8	8.16	63.40	1.327	6.194	0.647
		2013L	11.1	11.3	5.31	33.76	1.570	7.017	0.727
		2014E	18.3	18.2	7.86	54.29	1.783	7.230	0.712
	NN	2013E	50.5	46.3	17.73	97.24	0.399	1.379	0.714
		2013L	30.1	31.4	11.05	56.78	0.482	0.889	0.672
		2014E	41.0	38.0	15.58	82.67	1.109	2.228	0.713
HI	LN	2013E	0.55	0.53	0.10	0.78	−0.623	3.209	0.797
		2013L	0.60	0.61	0.28	0.74	−0.918	0.971	0.699
		2014E	0.61	0.59	0.23	0.72	−0.742	1.568	0.859
	NN	2013E	0.57	0.58	0.19	0.67	−0.744	0.590	0.858
		2013L	0.61	0.60	0.27	0.76	−0.361	1.339	0.700
		2014E	0.56	0.55	0.22	0.98	−0.398	3.721	0.929
TGW	LN	2013E	22.4	22.9	12.21	34.03	0.633	4.309	0.905
		2013L	19.7	20.4	13.40	28.39	0.750	1.775	0.889
		2014E	22.1	22.1	17.43	33.37	0.476	1.717	0.887
	NN	2013E	24.5	24.7	16.54	32.49	0.532	1.767	0.884
		2013L	21.7	21.3	15.65	31.70	0.567	1.114	0.859
		2014E	23.4	23.3	18.08	29.33	0.606	0.533	0.899
SF	LN	2013E	0.93	0.90	0.43	0.99	−2.192	8.976	0.874
		2013L	0.92	0.93	0.63	0.99	−1.512	3.147	0.796
		2014E	0.94	0.92	0.36	0.98	−1.383	3.360	0.849
	NN	2013E	0.94	0.94	0.10	0.99	−2.873	16.650	0.872
		2013L	0.95	0.96	0.52	0.99	−1.507	3.702	0.766
		2014E	0.93	0.83	0.49	0.98	−1.088	1.343	0.854

*^*a*^GY, grain yield per plant; BM, biomass per plant; HI, harvest index; TGW, 1000-grain weight; SF, spikelet fertility.*

*^*b*^LN, low nitrogen; NN, normal nitrogen; 2013E, early season of 2013; 2013L, late season of 2013; 2014E, early season of 2014.*

Two-way ANOVA (genotype and season) indicated that season was highly significant for GY, BM, HI, TGW, and SF in the two nitrogen treatments (Supplementary Table 1). Genotype was highly significant for GY, HI, TGW, and SF in the two nitrogen treatments. Genotype-by-season interaction was highly significant for HI and SF in the two nitrogen treatments. For the five traits, the main source of the variation was the season. The effect of genotype was significant for all five traits in the two conditions except for BM in the NN treatment.

In the LN, GY was highly, significantly, and positively correlated with other traits in three seasons except TGW in 2014E (Supplementary Table 2). In the NN, GY was highly significantly correlated with BM, HI, and SF, and not significantly correlated with TGW in the three seasons.

### QTL Mapping

**FIGURE 1:**
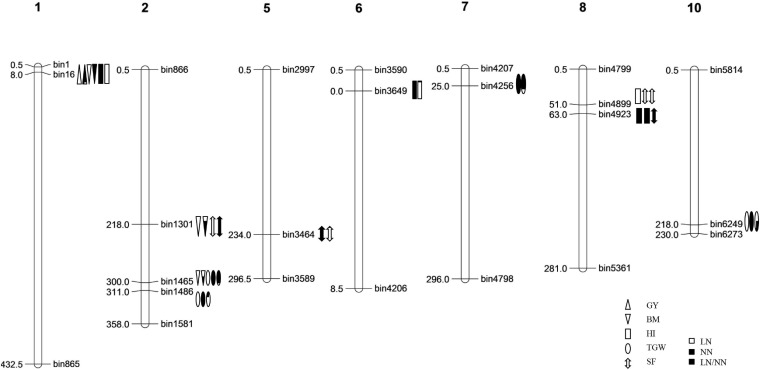
The genetic linkage map and the locations of QTLs for nitrogen deficiency tolerance (NDT)-related traits identified in the IB population.

A total of 40 QTLs were identified for the five NDT-related traits across the six environments (nitrogen–season combinations) (Table 2 and Figure 1).

**TABLE 2:** Putative QTLs for five NDT-related traits identified under LN, NN, and LN/NN conditions in three seasons.

Treatment^a^	Trait^b^	Season^c^	QTL	Chr.	Bin marker	Position (Mb)	LOD	R^2^ (%)
LN/NN	RGY	2013L	*qRGY1*	1	bin16	0.8	3.72	11.5
LN/NN	RBM	2013E	*qRBM2-1*	2	bin1301	21.8	4.19	4.8
LN/NN	RBM	2013E	*qRBM2-2*	2	bin1465	30.0	3.54	5.5
LN/NN	RHI	2013E	*qRHI1*	1	bin16	0.8	3.29	8.5
LN/NN	RHI	2013E	*qRHI6*	6	bin3649	3.0	8.25	20.1
LN/NN	RHI	2013E	*qRHI8*	8	bin4899	5.1	3.36	13.3
LN/NN	RTGW	2014E	*qRTGW2-1*	2	bin1465	30.0	6.51	19.6
LN/NN	RTGW	2014E	*qRTGW2-2*	2	bin1486	31.1	7.19	24.0
LN/NN	RTGW	2013E	*qRTGW7*	7	bin4256	2.5	4.39	6.1
LN/NN	RTGW	2014E	*qRTGW10*	10	bin6249	21.8	5.10	13.7
LN/NN	RSF	2014E	*qRSF5*	5	bin3464	23.4	3.20	7.9
LN/NN	RSF	2013E	*qRSF8*	8	bin4923	6.3	7.52	12.2
LN	GY	2013L	*qGY1*	1	bin16	0.8	5.21	15.0
LN	BM	2013L	*qBM1*	1	bin16	0.8	3.53	9.7
LN	BM	2013E	*qBM2-1*	2	bin1301	21.8	3.25	3.3
LN	BM	2013E	*qBM2-2*	2	bin1465	30.0	3.04	8.2
LN	HI	2013E	*qHI8-2*	8	bin4923	6.3	5.58	7.9
LN	TGW	2013E	*qTGW2-1*	2	bin1465	30.0	4.16	8.3
LN	TGW	2013L	*qTGW2-1*	2	bin1465	30.0	5.58	12.3
LN	TGW	2014E	*qTGW2-1*	2	bin1465	30.0	5.34	13.4
LN	TGW	2013L	*qTGW2-2*	2	bin1486	31.1	7.91	14.2
LN	TGW	2014E	*qTGW2-2*	2	bin1486	31.1	4.70	10.7
LN	TGW	2013L	*qTGW10*	10	bin6249	21.8	3.40	1.9
LN	TGW	2014E	*qTGW10*	10	bin6249	21.8	3.33	3.5
LN	SF	2013L	*qSF2*	2	bin1301	21.8	4.84	6.1
LN	SF	2013E	*qSF8*	8	bin4923	6.3	12.44	18.4
NN	BM	2014E	*qBM1*	1	bin16	0.8	3.39	8.3
NN	HI	2013E	*qHI1*	1	bin16	0.8	4.63	11.6
NN	HI	2013E	*qHI6-1*	6	bin3649	3.0	8.67	20.1
NN	HI	2014E	*qHI6-1*	6	bin3649	3.0	6.31	19.0
NN	HI	2013E	*qHI8-1*	8	bin4899	5.1	4.14	13.5
NN	TGW	2014E	*qTGW2-1*	2	bin1465	30.0	3.36	6.4
NN	TGW	2013L	*qTGW2-2*	2	bin1486	31.1	3.38	6.5
NN	TGW	2013E	*qTGW2-2*	2	bin1486	31.1	3.81	9.1
NN	TGW	2014E	*qTGW2-2*	2	bin1486	31.1	3.20	6.8
NN	TGW	2013E	*qTGW7*	7	bin4256	2.5	3.20	5.0
NN	TGW	2014E	*qTGW10*	10	bin6249	21.8	4.04	2.0
NN	SF	2013L	*qSF2*	2	bin1301	21.8	4.27	8.5
NN	SF	2013L	*qSF5*	5	bin3464	23.4	4.78	12.7
NN	SF	2013E	*qSF8-1*	8	bin4899	5.1	4.29	13.9

*^*a*^LN, low nitrogen; NN, normal nitrogen; LN/NN, ratio of LN to NN. ^*b*^GY, grain yield per plant; BM, biomass per plant; HI, harvest index; TGW, 1000-grain weight; SF, spikelet fertility; RGY, ratio of GY under LN to NN; RBM, ratio of BM under LN to NN; RHI, ratio of HI under LN to NN; RTGW, ratio of TGW under LN to NN; RSF, ratio of SF under LN to NN. ^*c*^2013E, early season of 2013; 2013L, late season of 2013; 2014E, early season of 2014.*

#### QTLs Detected for Trait Ratios of LN to NN (LN/NN)

For RGY, one QTL (*qRGY1*) was detected on chromosome 1 in 2013L and accounted for 11.5% of phenotypic variation (Table 2). For RBM, two QTLs (*qRBM2-1* and *qRBM2-2*) were identified on chromosome 2 in 2013E and explained 4.8% and 5.5% of the phenotypic variation, respectively. For RHI, three QTLs (*qRHI1*, *qRHI6*, and *qRHI8*) were identified in 2013E and distributed on chromosomes 1, 6, and 8 with the phenotypic variations of 8.5%, 20.1%, and 13.3%, respectively. Four QTLs for RTGW, including *qRTGW7* in 2013E, and *qRTGW2-1*, *qRTGW2-2*, and *qRTGW10* in 2014E, were detected on chromosomes 7, 2, 2, and 10, respectively, and explained 6.1%, 19.6%, 24.0%, and 13.7% of the phenotypic variations, respectively. For RSF, two QTLs (*qRSF5* and *qRSF8*) were identified on chromosomes 5 and 8 in 2014E and 2013E with phenotypic variations of 7.9% and 12.2%, respectively.

#### QTLs Detected for Traits in Low Nitrogen (LN) Condition

One QTL for GY, *qGY1* was detected on chromosome 1 in 2013L and explained 15.0% of the phenotypic variation (Table 2). For BM, three QTLs (*qBM1*, *qBM2-1*, and *qBM2-2*) were identified on chromosomes 1, 2, and 2 in 2013L and 2013E, and accounted for phenotypic variations of 9.7%, 3.3%, and 8.2%, respectively. One QTL named *qHI8-2* was identified for HI, which explained a phenotypic variation of 7.9%. Three QTLs were identified for TGW. Specifically, *qTGW2-1* was simultaneously detected on chromosome 2 in all three conditions with phenotypic variations from 8.3 to 13.4%. *qTGW2-2* was detected on chromosome 2 in 2013L and 2014E with phenotypic variations of 14.2% and 10.7%, while *qTGW10* was detected on chromosome 10 in 2013L and 2014E with phenotypic variations of 1.9–3.5%. Two QTLs (*qSF2*, *qSF8*) were mapped to chromosomes 2 and 8 for SF in 2013L and 2013E, which accounted for phenotypic variations of 6.1% and 18.4%, respectively.

#### QTLs Detected for Traits in Normal Nitrogen (NN) Condition

One QTL named *qBM1* was detected on chromosome 1 for BM in 2014E (Table 2), which explained 8.3% of the phenotypic variation. For HI, three QTLs (*qHI1*, *qHI6-1*, and *qHI8-1*) were identified. The QTL on chromosome 1, *qHI1*, was identified in 2013E and explained 11.6% of the phenotypic variation. The QTL, *qHI6-1*, on chromosome 6 was simultaneously identified in 2013E and 2014E and explained 20.1% and 19.0% of the phenotypic variation. The QTL *qHI8-1* on chromosome 8 was detected in 2013E and explained 13.5% of the phenotypic variation. Four QTLs were identified for TGW. *qTGW2-2* was simultaneously identified on chromosome 2 in all three conditions and explained 6.5–9.1% of phenotypic variation. *qTGW2-1* and *qTGW10* were mapped to chromosomes 2 and 10 in 2014E and explained 6.4% and 2.0% of the phenotypic variation, respectively. *qTGW7* was detected on chromosome 7 in 2013E with a phenotypic variation of 5.0%. For SF, two QTLs (*qSF2* and *qSF5*) were mapped on chromosomes 2 and 5 in 2013L with phenotypic variations of 8.5% and 12.7%, respectively, while another one (*qSF8-1*) was mapped on chromosome 8 in 2013E with phenotypic variations of 13.9%.

### Favorable Allele Mining of Important QTLs

Ten regions were detected for NDT-related traits in multiple seasons (Table 3). Among them, five were reported near the cloned genes related to nitrogen use efficiency (NUE) and/or functional ammonium transporter. The region bin16 on chromosome 1 was detected and associated with multiple traits, such as GY, RGY, BM, HI, and RHI, and the best favorable alleles all came from the donor parent OM1723 except the HI in 2013E NN environment. The region bin1301 was identified in relation to the traits BM, RBM, and SF, which were located very close to the cloned gene *LOC_Os02g38230* with functional annotation of partner protein for high-affinity nitrate transport (Liu et al., 2014). The best favorable alleles for BM and RBM came from the donor Teqing, while the best favorable alleles for SF came from OM1723 and Phalguna in 2013L LN and 2013L NN environments, respectively. Even though the best favorable alleles came from different donors, they are all carrying higher positive effects than the recurrent parent HHZ. The bin1465 and bin1486 were very near the cloned genes *LOC_Os02g47280* or *GRF4* (Hu et al., 2015; Li et al., 2018a) and *LOC_Os02g53130* (Gao et al., 2019) on chromosome 2 for NUE, respectively. At the region of bin1465, the favorable PSBRc28 alleles increased the traits RBM in 2013E LN/NN, BM in 2013E LN, and RTGW in 2014E LN/NN environments, whereas the favorable OM1723 alleles increased TGW in 2013E LN, 2013L LN, and 2014E NN environments. The bin1486 was detected in relation to TGW in both LN and NN environments with the favorable alleles coming from the donor OM1723. Bin3464, bin3649, bin4256, and bin4899 were detected for SF, HI, TGW, etc., in both NN and LN/NN environments with the favorable alleles coming from various donors. At the region of bin3464 harboring *LOC_Os05g39240* for functional ammonium transporter (Gaur et al., 2012), the favorable allele was from IR64 for RSF in 2014E LN/NN environment, while another one was from PSBRc28 for SF in 2013L NN environment. Similarly, at the region of bin3649, the favorable allele was from OM1723 for RHI in 2013E LN/NN, while the other ones were from PSBRc66 for HI in 2013E and 2014E NN environments. At the region of bin4256, the favorable OM1723 allele increased RTGW in 2013E LN/NN environment, while the favorable CDR22 allele increased TGW in 2013E NN environment. At the region of bin4899, the favorable alleles were from IR64, Teqing, and Phalguna for the traits RHI, HI, and SF in 2013E LN/NN, 2013E NN, and 2013E NN environments, respectively. The favorable PSBRc28 alleles at the bin4923 increased RSF in 2013E LN/NN and SF in 2013E LN environments, while the favorable Phalguna allele increased HI in 2013E LN environment. The bin6249 was detected for TGW and RTGW in LN, NN, and LN/NN environments of 2014E, with the favorable alleles all from Phalguna, which is located in the same region as the cloned gene *LOC_Os10g40600* with the functional annotation of nitrogen use efficiency (Hu et al., 2019; Zhang et al., 2019).

**TABLE 3:** Effects of putative QTLs identified in this study and the previously cloned genes related to nitrogen deficiency tolerance.

Bin marker	Chr	Physical position (kb)	Treatment^a^	Season^b^	QTL	Allelic effect	Cloned gene	Annotation
bin16	1	750–800	LN/NN	2013L	*qRGY1*	OM1723 (0.013) > HHZ (−0.004)		
			LN	2013L	*qGY1*	OM1723 (1.437) > HHZ (−0.427)		
			LN	2013L	*qBM1*	OM1723 (2.512) > HHZ (−0.996)		
			NN	2014E	*qBM1*	OM1723 (0.996) > HHZ (−0.798)		
			LN/NN	2013E	*qRHI1*	OM1723 (0.211) > HHZ (−0.087)		
			NN	2013E	*qHI1*	HHZ (0.054) > OM1723 (−0.051)		
bin1301	2	21750–21800	LN/NN	2013E	*qRBM2-1*	Teqing (0.184) > IR64 (0.035) > HHZ (−0.004) > OM1723 (−0.051) > PSBRc66 (−0.052) > Phalguna (−0.062) > CDR22 (−0.064)	*LOC_Os02g38230* (Liu et al., 2014)	Partner protein for high- affinity nitrate transport
			LN	2013E	*qBM2-1*	Teqing (5.041) > IR64 (3.529) > HHZ (−0.115) > CDR22 (−1.321) > PSBRc66 (−1.651) > OM1723 (−1.864) > Phalguna (−4.711)		
			LN	2013L	*qSF2*	OM1723 (0.038) > IR64 (0.029) > CDR22 (0.023) > HHZ (0.018) > Teqing (−0.017) > Phalguna (−0.037) > PSBRc66 (−0.11)		
			NN	2013L	*qSF2*	Phalguna (0.071) > OM1723 (0.032) > HHZ (0.028) > IR64 (0.021) > CDR22 (0.001) > Teqing (−0.059) > PSBRc66 (−0.16)		
bin1465	2	29950–30000	LN/NN	2013E	*qRBM2-2*	PSBRc28 (0.355) > Teqing (0.027) > HHZ (−0.008) > IR64 (−0.009) > IR50 (−0.017) > PSBRc66 (−0.062) > OM1723 (−0.07)	*LOC_Os02g47280* (Hu et al., 2015; Li et al., 2018a)	Growth-regulating factor; nitrogen use efficiency
			LN	2013E	*qBM2-2*	PSBRc28 (8.816) > IR64 (1.364) > Teqing (0.693) > HHZ (−0.401) > IR50 (−0.484) > PSBRc66 (−2.277) > OM1723 (−2.473)		
			LN/NN	2014E	*qRTGW2-1*	PSBRc28 (0.04) > Teqing (0.003) > PSBRc66 (0.001) > HHZ (−0.007) > OM1723 (−0.013) > IR50 (−0.02) > IR64 (−0.026)		
			LN	2013E	*qTGW2-1*	OM1723 (1.705) > PSBRc66 (1.391) > PSBRc28 (0.77) > HHZ (−0.265) > IR64 (−0.695) > IR50 (−1.044) > Teqing (−1.449)		
			LN	2013L	*qTGW2-1*	OM1723 (1.11) > PSBRc66 (0.807) > IR64 (0.111) > PSBRc28 (−0.036) > IR50 (−0.444) > HHZ (−0.649) > Teqing (−1.295)		
			LN	2014E	*qTGW2-1*	PSBRc66 (1.7) > OM1723 (1.32) > PSBRc28 (1.121) > HHZ (−0.379) > IR64 (−0.881) > Teqing (−1.132) > IR50 (−1.589)		
			NN	2014E	*qTGW2-1*	OM1723 (1.766) > PSBRc66 (1.648) > IR64 (−0.034) > PSBRc28 (−0.121) > HHZ (−0.175) > IR50 (−1.007) > Teqing (−1.211)		
bin1486	2	31000–31050	LN/NN	2014E	*qRTGW2-2*	Phalguna (0.017) > PSBRc66 (0.006) > HHZ (−0.001) > OM1723 (−0.019) > IR64 (−0.026)	*LOC_Os02g53130* (Gao et al., 2019)	Nitrogen use efficiency
			LN	2013L	*qTGW2-2*	OM1723 (2.052) > PSBRc66 (0.683) > IR64 (−0.021) > Phalguna (−0.446) > HHZ (−1.123)		
			NN	2013L	*qTGW2-2*	OM1723 (2.139) > PSBRc66 (0.503) > IR64 (0.334) > Phalguna (−0.6) > HHZ (−0.92)		
			NN	2013E	*qTGW2-2*	OM1723 (1.772) > PSBRc66 (0.805) > Phalguna (0.13) > IR64 (−0.795) > HHZ (−0.988)		
			LN	2014E	*qTGW2-2*	OM1723 (1.256) > PSBRc66 (1.07) > Phalguna (0.318) > HHZ (−1.104) > IR64 (−1.459)		
			NN	2014E	*qTGW2-2*	OM1723 (1.947) > PSBRc66 (0.832) > Phalguna (−0.148) > IR64 (−0.644) > HHZ (−1.103)		
bin3464	5	23350–23400	LN/NN	2014E	*qRSF5*	IR64 (0.04) > Teqing (0.025) > HHZ (0.009) > CDR22 (−0.001) > PSBRc66 (−0.007) > PSBRc28 (−0.009)	*LOC_Os05g39240* (Gaur et al., 2012)	Functional ammonium transporter, constitutively expressed in roots and shoots
			NN	2013L	*qSF5*	PSBRc28 (0.035) > HHZ (0.024) > IR64 (0.018) > CDR22 (0.008) > Teqing (−0.014) > PSBRc66 (−0.118)		
bin3649	6	2950–3000	LN/NN	2013E	*qRHI6*	OM1723 (0.336) > HHZ (−0.082) > PSBRc66 (−0.123)		
			NN	2013E	*qHI6-1*	PSBRc66 (0.048) > HHZ (0.028) > OM1723 (−0.105)		
			NN	2014E	*qHI6-1*	PSBRc66 (0.043) > HHZ (0.034) > OM1723 (−0.083)		
bin4256	7	2450–2500	LN/NN	2013E	*qRTGW7*	OM1723 (0.034) > Phalguna (0.022) > CDR22 (0.01) > PSBRc66 (0.007) > IR64 (0.004) > Teqing (−0.007) > HHZ (−0.008) > IR50 (−0.012)		
			NN	2013E	*qTGW7*	CDR22 (1.527) > OM1723 (0.532) > HHZ (0.186) > PSBRc66 (−0.037) > IR64 (−0.137) > IR50 (−0.687) > Phalguna (−0.747) > Teqing (−1.011)		
bin4899	8	5000–5050	LN/NN	2013E	*qRHI8*	IR64 (0.138) > OM1723 (0.049) > Phalguna (−0.047) > HHZ (−0.049) > Teqing (−0.066) > PSBRc66 (−0.084) > CDR22 (−0.09)		
			NN	2013E	*qHI8-1*	Teqing (0.054) > Phalguna (0.027) > OM1723 (0.021) > PSBRc66 (0.018) > HHZ (0.017) > CDR22 (0.007) > IR64 (−0.094)		
			NN	2013E	*qSF8-1*	Phalguna (0.039) > PSBRc66 (0.035) > HHZ (0.019) > CDR22 (0.011) > Teqing (0.003) > OM1723 (0.001) > IR64 (−0.104)		
bin4923	8	6200–6250	LN/NN	2013E	*qRSF8*	PSBRc28 (0.204) > IR50 (0.083) > IR64 (0.061) > HHZ (−0.001) > CDR22 (−0.009) > Phalguna (−0.015) > PSBRc66 (−0.02) > OM1723 (−0.43)		
			LN	2013E	*qSF8-2*	PSBRc28 (0.07) > IR50 (0.06) > PSBRC66 (0.06) > Phalguna (0.044) > HHZ (0.036) > CDR22 (0.026) > IR64 (−0.04) > OM1723 (−0.37)		
			LN	2013E	*qHI8-2*	Phalguna (0.046) > HHZ (0.041) > IR50 (0.026) > PSBRc66 (0.026) > CDR22 (0.004) > IR64 (−0.023) > PSBRc28 (−0.064) > OM1723 (−0.204)		
bin6249	10	21750–21800	LN/NN	2014E	*qRTGW10*	Phalguna (0.029) > HHZ (0.005) > PSBRc66 (−0.002) > OM1723 (−0.008) > IR50 (−0.008)	*LOC_Os10g40600* (Hu et al., 2019; Zhang et al., 2019)	Nitrogen use efficiency
			LN	2013L	*qTGW10*	PSBRc66 (2.174) > Phalguna (1.672) > OM1723 (1.308) > HHZ (−1.225) > IR50 (−1.248)		
			LN	2014E	*qTGW10*	Phalguna (3.728) > OM1723 (1.872) > HHZ (−0.662) > PSBRc66 (−0.849) > IR50 (−1.692)		
			NN	2014E	*qTGW10*	Phalguna (2.747) > OM1723 (2.184) > HHZ (−0.826) > PSBRc66 (−0.836) > IR50 (−1.353)		

*^*a*^LN, low nitrogen; NN, normal nitrogen; LN/NN, ratio of LN to NN.*

*^*b*^2013E, early season of 2013; 2013L, late season of 2013; 2014E, early season of 2014.*

### Selecting Super Nitrogen Deficiency Tolerant Lines

Based on the favorable allele dissection, six superior lines with four favorable introgressed alleles were selected (Table 4). Compared with the recurrent parent HHZ, the six lines all showed significantly higher GY in LN environments and similar GY under NN environments except for H10. Out of the six lines, Line H11 carrying simultaneously three detected QTLs for GY and TGW with the best favorable alleles all coming from OM1723 performed the highest GY, TGW, and BM in LN environments. Lines H10 and H12, separately carrying QTLs underlying GY and TGW, were superior to HHZ in terms of GY, TGW, and BM in LN environments. Three lines (H289, H456, and H53) harbored different QTLs affecting BM wherein the best favorable alleles were from Teqing, PSBRc28, and OM1723, respectively. The above six lines carrying different favorable introgressed alleles for NDT-related traits from various donors are beneficial for pyramid breeding for improved GY under LN environment.

**TABLE 4:** Phenotypic values and favorable introgressed alleles of the selected six superior lines.

Line	Envi.^a^	GY^b^	TGW	Biomass	Bin16	Bin1301	Bin1465	Bin1486
H10	LN	11.00 ± 1.19*	26.21 ± 1.06**	20.89 ± 2.68*	2013L_LN_GY (OM1723) ^*c*^ 2013L_ LN/NN _GY (OM1723)	–	2013E_LN_TGW (OM1723) 2013L_LN_TGW (OM1723) 2014E_NN_TGW (OM1723)	–
	NN	15.98 ± 2.84*	28.13 ± 1.27**	42.72 ± 7.19		–		–
H11	LN	14.63 ± 5.9**	26.34 ± 1.1**	27.04 ± 11.3**		–		2013L_LN_TGW (OM1723) 2014E_LN_TGW (OM1723) 2013L_NN_TGW (OM1723) 2014E_NN_TGW (OM1723)
	NN	17.82 ± 2.45	27.57 ± 1.28**	42.63 ± 5.99		–		
H12	LN	12.43 ± 4.04*	25.66 ± 1.7**	25.10 ± 10.53*	–	–		–
	NN	20.21 ± 4.33	27.62 ± 1.23**	44.64 ± 2.43	–	–		–
H289	LN	11.36 ± 3.93*	20.93 ± 1.88	22.78 ± 12.05	–	2013E_LN_BM (Teqing) 2013E_ LN/NN _BM (Teqing)	–	–
	NN	20.81 ± 3.88	22.39 ± 1.44	36.71 ± 7.04	–		–	–
H456	LN	10.82 ± 4.32*	22.54 ± 1.34	19.53 ± 7.16	–	–	2013E_LN_BM (PSBRc28) 2013E_ LN/NN _BM (PSBRc28)	–
	NN	22.67 ± 9.12	23.36 ± 0.92	41.64 ± 13.16	–	–		–
H53	LN	13.02 ± 1.64*	23.95 ± 0.07**	24.34 ± 2.6**	2013L_LN_BM (OM1723) 2014E_NN_BM (OM1723)	–	–	–
	NN	17.31 ± 1.67	25.08 ± 0.38*	42.00 ± 7.23		–	–	–
HHZ (CK)	LN	8.42 ± 1.62	21.46 ± 1.17	15.75 ± 3.51	–	–	–	–
	NN	20.17 ± 2.22	23.09 ± 1.31	38.84 ± 6.44	–	–	–	–

^*a*^LN, low nitrogen; NN, normal nitrogen; LN/NN, ratio of LN to NN. ^*b*^Data of GY (grain yield per plant) and TGW (1000-grain weight) were calculated based on the three seasons. * and ** represent significant levels at *P* < 0.05 and 0.01, respectively. ^*c*^The environment where the bins were detected; the parents in the brackets are those whose favorable alleles came from.

### Fine Mapping of *qTGW2-1*

**FIGURE 2:**
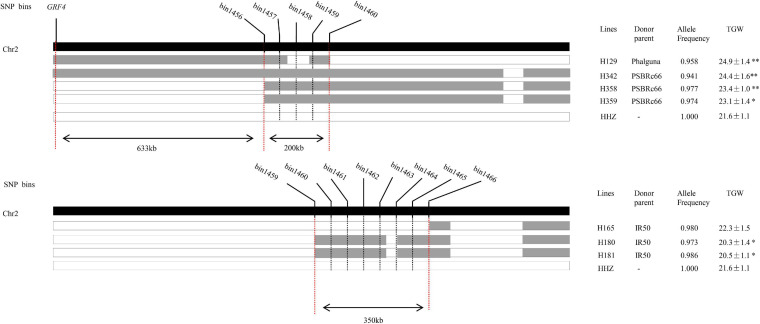
The physical location of *qTGW2-1* delimited by graphical genotype mapping. The black-colored strip indicates chromosome 2. The gray and white strips show the homozygous introgressed segment derived from the donor and HHZ, respectively. The allele frequency is the percentage of the alleles of HHZ. * and ** represent significant differences at *P* ≤ 0.05 and 0.01, respectively.

The *qTGW2-1*, which was simultaneously detected in 2014E in three conditions (LN, NN, and LN/NN) and also in 2013E and 2013L in LN conditions, most likely associated with NDT and the favorable alleles mainly came from OM1723 and PSBRc28 in different conditions. To fine map the stable *qTGW2-1* in the region of bin1456–bin1466 on chromosome 2 by means of substitution mapping, ILs with overlapped introgressed segments and covered the genomic region of *qTGW2-1* were selected from the IB population. Seven selected ILs had the recurrent genome percentage higher than 94.0%. The graphic genotypes of these seven ILs are given in Figure 2. Three, three, and one lines had the alleles from the donor parents PSBRc66, IR50, and Phalguna, respectively. The lines of H129, H342, H358, and H359 had significantly higher TGW than the recipient parent HHZ, while H180 and H181 had significantly lower TGW than the recipient parent. The TGW of H165 was very similar to that of HHZ. Therefore, the *qTGW2-1* was separated into two QTLs for TGW, one was located in about the 200-kb region flanked by bin1456 and bin1460 with the positive alleles for increased TGW from the donor parent PSBRc66 and Phalguna, and the other one was located in about the 350-kb region delimited by bin1459 and 1466 with the negative allele for decreased TGW from the donor parent IR50. The *GRF4* for NUE (Hu et al., 2015; Li et al., 2018a) was around 633-kb apart from the QTL flanked by bin1456 and bin1460 (Figure 2), suggesting the two QTLs separated from *qTGW2-1* were new loci for NDT.

## Discussion

### Comparisons of the NDT QTLs Identified in This Study With Previously Reported QTLs and Cloned Genes

Nitrogen-deficiency tolerance (NDT) traits were measured by the ratios of the trait values under LN to those under NN and could be used as a criterion for selecting genotypes for the tolerance of low nitrogen. Many agronomic traits such as effective tiller number, biomass, and grain yield have been studied under low-N stress in rice, and some QTLs associated with N translocation were found to locate in the same chromosomal regions (Shan et al., 2005; Tong et al., 2006; Senapathy et al., 2008). Among 10 QTL regions identified for the NDT-related traits in this study (Table 3), the region bin16 on chromosome 1 harboring QTLs for BM, GY, and HI was associated with the QTLs affecting shoot dry weight and plant dry weight at seedling stage under low nitrogen condition identified by Lian et al. (2005), and the QTLs for relative shoot weight, relative plant height, and relative biomass yield at seedling stage (Feng et al., 2010). QTLs affecting BM and SF in the bin1301 identified in this study were mapped together with the QTLs for dry shoot weight under low nitrogen condition (Lian et al., 2005), grain nitrogen use efficiency and grain yield (Wei et al., 2011), soluble protein content detected in the backcross inbred lines of Nipponbare/Kasalath (Ishimaru et al., 2001), and the cloned gene *LOC_Os02g38230* for partner protein for high-affinity nitrate transport (Liu et al., 2014). The region bin1465 harboring QTLs for BM and for TGW on chromosome 2 identified in the present study were mapped together with the QTL affecting root dry weight under low nitrogen condition (Lian et al., 2005) and near the *LOC_Os02g47280* for nitrogen use efficiency and growth-regulating factor (Hu et al., 2015; Li et al., 2018a), and the *gy2b* affecting GY under low nitrogen condition (Cho et al., 2007). The bin1486 harboring QTLs for TGW and RTGW on chromosome 2 in this study were mapped together to the QTL for the ratio of plant height under low nitrogen stress to normal conditions (Feng et al., 2010) and the *LOC_Os02g53130*, a nitrate reductase gene for nitrogen use efficiency (Gao et al., 2019). The bin3464 harboring QTLs for RSF and SF on chromosome 5 in this study was mapped near the *qHGW-5b* for grain weight under low nitrogen input (Tong et al., 2011) and the *LOC_Os05g39240* for functional ammonium transporter, which was constitutively expressed in roots and shoots (Gaur et al., 2012). The bin4899 harboring QTLs for HI and SF on chromosome 8 in this study was associated with the QTL for plant dry weight under low nitrogen condition (Lian et al., 2005). The bin6249 on chromosome 10 affecting TGW detected under LN, NN, and LN/NN conditions in this study harbored the QTL (*qFGPP-10b*) for filled grains per panicle under low nitrogen input (Tong et al., 2011), the *qNR10* for nitrogen response (Wei et al., 2012), and the *LOC_Os10g40600*, a nitrate-transporter gene for nitrogen use efficiency (Hu et al., 2019; Zhang et al., 2019). Allelisms of the above QTLs for NDT-related traits identified in this study with the previously reported QTLs or cloned genes for NDT or NUE require further verification via fine mapping and QTL cloning.

Nitrogen deficiency tolerance is defined as the plants’ ability to maintain normal growth and good yield when the soil N content is low. Thus, NDT could have something to do with NUE to some extent. Based on comparisons of QTLs affecting NDT detected in this study with those previously reported, five regions underlying NDT at bins 1301, 1465, 1486, 3464, and 6249 on chromosomes 2, 2, 2, 5, and 10, respectively, were found to map together with or near the five cloned NUE-related genes. Using 127 recombinant inbred lines (RILs) derived from the cross of Zhenshan97/Minghui63, Wei et al. (2012) also identified four genomic regions containing QTLs for NDT and NUE traits with the same additive effect directions. It was suggested that partial genetic overlaps at least exist between NDT and NUE, which is beneficial for rice breeding for simultaneously improving NDT and NUE.

### Mining Favorable Alleles for NDT

From the breeding point of view, the major use of identified QTL or cloned gene is to mine favorable alleles for efficient molecular breeding. So far, over 2,000 genes controlling important agronomic traits have been cloned in rice (Li et al., 2018b). Unfortunately, the phenotypic effects of different alleles in rice germplasm accessions at these cloned rice genes and their values in rice breeding remain largely unknown. This is one of the most important reasons why so many cloned genes have never been exploited in breeding through MAS so far. Using eight BC1 populations derived from a widely adaptable recipient and eight donors plus three rounds of phenotypic selection, 496 trait-specific introgression lines (ILs) in Huanghuazhan (HHZ) background have been developed (Ali et al., 2017), forming a material platform for discovery of QTLs underlying the target and non-target traits (Zhu et al., 2015; Feng et al., 2018). The IL population has characteristics of similar developmental stage and plant height in elite rice background, thus very suitable for evaluation of abiotic stress tolerances such as drought, salt, and cold whose tolerance performances largely depend on developmental stage. In this study, favorable alleles at QTLs for different NDT-related traits in the 10 NDT-related regions were dissected (Table 3). Among the total 40 loci affecting different NDT-related traits at the 10 bins under different conditions (Table 3), frequencies of favorable alleles were 16 for OM1723, seven for Phalguna, six for PSBRc28, four for PSBRc66, three for Teqing, two for IR64, and one for each of CDR22 and HHZ. Obviously, OM1723, an *indica* variety from Vietnam is an outstanding parent with many favorable alleles for improving NDT. For instance, the OM1723 alleles simultaneously increased GY under LN and LN/NN, BM under LN and NN, and RHI under LN/NN for all QTLs except *qHI1* at bin16 under NN, and TGW at almost all QTLs except *qRTGW2-2* at bin1486 under LN/NN. Similarly, the Phalguna alleles improved TGW for all QTLs except *qTGW10* at bin6249 under LN in 2013L. So, the bin16 is one of the most important regions for rice breeding of NDT. Information of favorable allele dissection for different NDT QTLs will be beneficial for introgressing and pyramiding breeding to improve NDT by MAS.

### Implications for Breeding

Historically, many breeding programs took yield potential as a primary target, particularly in China in the past few decades. In China, pursuing high yield of super rice cultivar has been accompanied with excess uses of chemical fertilizers and pesticides in rice production, resulting in serious environmental pollutions and largely reduced rate of fertilizer utilization by the crops (Zhang, 2007). Nevertheless, modern semidwarf rice cultivars have rarely achieved their yield potentials in farmers’ fields because of many abiotic and biotic stresses. The main reason for this huge yield gap is primarily due to the fact that most super rice cultivars developed under the high input conditions do not perform well in more than 70% of the moderate- and low-yielding fields in China, much of which suffer more frequently inadequate fertilizers and other abiotic stresses (Pandey, 1997; Feng et al., 2018). Consequently, developing crops that are less dependent on the heavy application of N fertilizers is essential for the sustainability of agriculture. NDT traits have been considered as indirect selection criteria for the improvement of NUE (Lian et al., 2005; Namai et al., 2009; Feng et al., 2010; Wei et al., 2012). As a matter of experience, the lines with superior yield and yield-related traits under both LN and NN conditions, or superior under LN and normal or average under NN, could be preferentially selected because such lines can use the nitrogen efficiently to produce BM and GY under limited nitrogen supply (Lafitte and Edmeades, 1994).

Nitrogen deficiency tolerance and NUE are complex quantitative traits controlled by multiple genes, which makes it difficult to improve these complex traits using conventional breeding approaches. Recently, high-throughput SNP genotyping based on re-sequencing and gene chips promises to greatly accelerate QTL mapping and pyramiding on the whole genome (Thomson, 2014; Feng et al., 2018). Ten important NDT regions were identified in the breeding population with elite variety background in this study. Some of them were co-located with the previously reported NUE-related QTLs or genes, which are useful for rice breeding for high NDT or both for NDT and NUE by introgressing or pyramiding of favorable alleles at those important loci by MAS. Specifically, the Bin16 affecting GY, RGY, BM, and RHI under different nitrogen conditions, and other five bins 1301, 1465, 1486, 3464, and 6249 shared with the previously reported QTLs, or genes underlying NDT- and NUE-related traits could be used in MAS-based or QTL-designed breeding for enhancing NDT and/or NUE in rice. As indicated in Table 4, two promising NDT lines, H10 carrying favorable alleles from OM1723 at bins 16 and 1465, and H11 with favorable alleles from OM1723 at bins 16, 1465, and 1486, can be used as donor parents to improve NDT of an elite variety by marker-assisted selection against the flanking markers linked to respective bins. Of course, important NDT loci identified herein could be also pyramided with other previously identified QTLs or genes controlling NDT and NUE as most of the NDT and NUE QTLs identified previously under different environmental conditions were genetically independent (Lian et al., 2005; Senapathy et al., 2008).

## Conclusion

A total of 14, 14, and 12 QTLs for the five traits was identified under LN, NN, and LN/NN conditions, respectively, in an interconnected breeding population across three seasons. Among them, 10 NDT-bin regions were identified, and the favorable alleles contributing to NDT-related traits were primarily from OM1723, secondarily from Phalguna. Six superior lines showed significantly higher GY in LN environments and similar GY under NN environments due to introgressing the favorable alleles at the related NDT-QTLs. The bins 16, 1301, 1465, 1486, 3464, and 6249 shared the QTLs or genes for NDT identified in this study and NUE previously reported could be used for improvement of NDT and NUE by MAS.

## Data Availability Statement

All datasets presented in this study are included in the article/Supplementary Material.

## Author Contributions

JX and ZL designed the experiment. CS, KC, JC, SZ, and YZ performed all the phenotypic evaluation. XM and YC performed the analysis and interpretation of the data. JX, XM, and YC drafted the manuscript. GY and JA revised the manuscript. All authors contributed to the article and approved the submitted version.

## Conflict of Interest

The authors declare that the research was conducted in the absence of any commercial or financial relationships that could be construed as a potential conflict of interest.

## Footnotes

1 https://github.com/JulongWei/MagicQTL

## References

[B1] Ali A. J. Xu J. L. Ismail A. M. Fu B. Y. Vijayakumar C. H. M. Gao Y. M. (2006). Hidden diversity for abiotic and biotic stress tolerances in the primary gene pool of rice revealed by a large backcross breeding program. *Field Crop Res.* 97 66–76. 10.1016/j.fcr.2005.08.016

[B2] Ali J. Xu J. L. Gao Y. M. Ma X. F. Meng L. J. Wang Y. (2017). Harnessing the hidden genetic diversity for improving multiple abiotic stress tolerance in rice (Oryza sativa L.). *PLoS One* 12:e0172515. 10.1371/journal.pone.0172515 PMC5344367 28278154

[B3] Buckler E. S. (2009). The genetic architecture of maize flowering time. *Science* 325 714–718. 10.1126/science.1174276 19661422

[B4] Cavanagh C. R. Morell M. K. Mackay I. Powell W. (2008). From mutations to MAGIC: resources for gene discovery, validation and delivery in crop plants. *Curr. Opin. Plant Biol.* 11 215–221. 10.1016/j.pbi.2008.01.002 18295532

[B5] Chen H. W. Zhao X. Q. Zhai L. Y. Shao K. T. Jiang K. J. Shen C. C. (2020). Genetic bases of the stomata-related traits revealed by a genome-wide association analysis in rice (Oryza sativa L.). *Front. Genet.* 11:611. 10.3389/fgene.2020.00611 32582301 PMC7296080

[B6] Cho Y. I. Jiang W. Z. Chin J. H. Piao Z. Z. Cho Y. G. McCouch S. R. (2007). Identification of QTLs associated with physiological nitrogen use efficiency in rice. *Mol. Cells.* 23 72–79. 17464214

[B7] Cook J. P. McMullen M. D. Holland J. B. Tian F. Bradbury P. J. Ross-Ibarra J. (2012). Genetic architecture of maize kernel composition in the nested association mapping and inbred association panels. *Plant Physiol.* 158 824–834. 10.1104/pp.111.185033 22135431 PMC3271770

[B8] Cui Y. R. Zhang F. Xu J. L. Li Z. K. Xu S. Z. (2015). Mapping quantitative trait loci in selected breeding populations: a segregation distortion approach. *Heredity* 115 538–546. 10.1038/hdy.2015.56 26126541 PMC4806901

[B9] Feng B. Chen K. Cui Y. R. Wu Z. C. Zheng T. Q. Zhu Y. J. (2018). Genetic dissection and simultaneous improvement of drought and low nitrogen tolerances by designed QTL pyramiding in rice. *Front. Plant Sci.* 9:306. 10.3389/fpls.2018.00306 29593764 PMC5855007

[B10] Feng Y. Cao L. Y. Wu W. M. Shen X. H. Zhan X. D. Zhai R. R. (2010). Mapping QTLs for nitrogen-deficiency tolerance at seedling stage in rice (Oryza sativa L.). *Plant Breed.* 129 652–656. 10.1111/j.1439-0523.2009.01728.x

[B11] Gao Z. Y. Wang Y. F. Chen G. Zhang A. Yang S. L. Shang L. G. (2019). The indica nitrate reductase gene OsNR2 allele enhances rice yield potential and nitrogen use efficiency. *Nat. Commun.* 10:5207. 10.1038/s41467-019-13110-8 31729387 PMC6858341

[B12] Gaur V. S. Singh U. S. Gupta A. K. Kumar A. (2012). Influence of different nitrogen inputs on the members of ammonium transporter and glutamine synthetase genes in two rice genotypes having differential responsiveness to nitrogen. *Mol. Biol. Rep.* 39 8035–8044. 10.1007/s11033-012-1650-8 22531935

[B13] He Y. X. Zheng T. Q. Hao X. B. Wang L. F. Gao Y. M. Hua Z. T. (2010). Yield performances of japonica introgression lines selected for drought tolerance in a BC breeding programme. *Plant Breed.* 129 167–175. 10.1111/j.1439-0523.2009.01683.x

[B14] Hu B. Jiang Z. Wang W. Qiu Y. Zhang Z. Liu Y. (2019). Nitrate–NRT1.1B–SPX4 cascade integrates nitrogen and phosphorus signaling networks in plants. *Nat. Plants* 5 401–413. 30911122 10.1038/s41477-019-0384-1

[B15] Hu J. Wang Y. X. Fang Y. X. Zeng L. J. Xu J. Yu H. P. (2015). A rare allele of GS2 enhances grain size and grain yield in rice. *Mol. Plan.* 8 1146–1155. 10.1016/j.molp.2015.07.002 26187814

[B16] Huang X. Feng Q. Qian Q. Zhao Q. Wang L. Wang A. (2009). High-throughput genotyping by whole-genome resequencing. *Genome Res.* 19 1068–1076. 19420380 10.1101/gr.089516.108 PMC2694477

[B17] Ishimaru K. Yano M. Aoki N. Ono K. Hirose T. Lin S. Y. (2001). Toward the mapping of physiological and agronomic characters on a rice function map. *Theor. Appl. Genet.* 102 793–800. 10.1007/s001220000467

[B18] Jordan D. R. Mace E. S. Cruickshank A. W. Hunt C. H. Henzell R. G. (2011). Exploring and exploiting genetic variation from unadapted sorghum germplasm in a breeding program. *Crop Sci.* 51 1444–1457. 10.2135/cropsci2010.06.0326

[B19] Kump K. L. Bradbury P. J. Wisser R. J. Buckler E. S. Belcher A. R. Oropeza-Rosas M. A. (2011). Genome-wide association study of quantitative resistance to southern leaf blight in the maize nested association mapping population. *Nat. Genet.* 43 163–168. 10.1038/ng.747 21217757

[B20] Lafitte H. R. Edmeades G. O. (1994). Improvement for tolerance to low soil nitrogen in tropical maize. II. Grain yield, biomass production, and N accumulation. *Field Crops Res.* 39 15–25. 10.1016/0378-4290(94)90067-1

[B21] Li S. Tian Y. Wu K. Ye Y. Yu J. Zhang J. (2018a). Modulating plant growth–metabolism coordination for sustainable agriculture. *Nature* 560 595–600. 30111841 10.1038/s41586-018-0415-5 PMC6155485

[B22] Li Y. Xiao J. H. Chen L. L. Huang X. H. Cheng Z. K. Han B. (2018b). Rice functional genomics research: past decade and future. *Mol. Plant* 11 359–380. 29409893 10.1016/j.molp.2018.01.007

[B23] Li Z. K. Fu B. Y. Gao Y. M. Xu J. L. Ali J. Lafitte H. R. (2005). Genome-wide introgression lines and their use in genetic and molecular dissection of complex phenotypes in rice (Oryza sativa L.). *Plant Mol. Biol.* 59 33–52. 10.1007/s11103-005-8519-3 16217600

[B24] Lian X. M. Xing Y. Z. Yan H. Xu C. G. Li X. H. Zhang Q. F. (2005). QTLs for low nitrogen tolerance at seedling stage identified using a recombinant inbred line population derived from an elite rice hybrid. *Theor. Appl. Genet.* 112 85–96. 16189659 10.1007/s00122-005-0108-y

[B25] Liu X. Q. Huang D. M. Tao J. Y. Miller A. J. Fan X. R. Xu G. H. (2014). Identification and functional assay of the interaction motifs in the partner protein OsNAR2.1 of the two-component system for high-affinity nitrate transport. *New Phytol.* 204 74–80. 25103875 10.1111/nph.12986 PMC4232926

[B26] Lv H. X. Zhou G. S. Ding Z. H. Sun Y. J. Yu S. B. (2010). QTL Identification for nitrogen responses in rice chromosomal segment substitution lines. *Mol. Plant Breed.* 8 1074–1081.

[B27] Meng L. J. Lin X. Y. Wang J. M. Chen K. Cui Y. R. Xu J. (2013). Simultaneous improvement of cold tolerance and yield of temperate japonica rice (Oryza sativa L.) by introgression breeding. *Plant Breed.* 132 604–612. 10.1111/pbr.12097

[B28] Namai S. Toriyama K. Fukuta Y. (2009). Genetic variation in dry matter production and physiological nitrogen use efficien-cy in rice (Oryza sativa L.) varieties. *Breed Sci.* 59 269–276.

[B29] Pandey S. (1997). “Rainfed lowland rice research: challenges and priorities for the 21st century,” in *Breeding Strategies for Rainfed Lowland Rice in Drought-prone Environments*, eds Fukai S. Cooper M. D. Salisbury J. (Canberra: Australian Centre for International Agricultural Research), 1–12.

[B30] Qu P. P. Shi J. H. Chen T. X. Chen K. Shen C. C. Wang J. K. (2020). Construction and integration of genetic linkage maps from three multi-parent advanced generation inter-cross populations in rice. *Rice* 13:13. 10.1186/s12284-020-0373-z 32060661 PMC7021868

[B31] Senapathy S. Kunnummal K. V. Palaniappan M. Marappa M. (2008). QTL and QTL× environment effects on agronomic and nitrogen acquisition traits in rice. *J. Integr. Plant Biol.* 50 1108–1117. 10.1111/j.1744-7909 2008.00713.x 18844779

[B32] Shan Y. H. Wang Y. L. Pan X. B. (2005). Mapping of QTLs for nitrogen use efficiency and related traits in rice. *Sci. Agric. Sinica* 4 721–727.

[B33] Tang J. Y. Zhang T. Jiang K. F. Yang L. Yang Q. H. Wan X. Q. (2011). Identification of QTL for yield traits of low nitrogen stress by using introgression lines of rice. *J. Agric. Biotech.* 19 996–1002.

[B34] Thomson M. J. (2014). High-throughput SNP genotyping to accelerate crop improvement. *Plant Breed. Biotechnol.* 2 195–212. 10.9787/PBB.2014.2.3.195

[B35] Tian F. Bradbury P. J. Brown P. J. Hung H. Y. Sun Q. Flintgarcia S. (2015). Genome-wide association study of leaf architecture in the maize nested association mapping population. *Nat. Genet.* 43 159–162. 10.1038/ng.746 21217756

[B36] Tong H. H. Chen L. Li W. P. Xing Y. Z. Yu X. Q. Xu X. Y. (2011). Identification and characterization of quantitative trait loci for grain yield and its components under different nitrogen fertilization levels in rice (Oryza sativa L.). *Sci. Agric. Sinica* 28 495–509. 10.1007/s11032-010-9499-9

[B37] Tong H. H. Mei H. W. Yu X. Q. Xu X. Y. Li M. S. Zhang S. Q. (2006). Identification of related QTLs at late developmental stage in rice (Oryza sativa L.) under two nitrogen levels. *Acta Genet. Sin.* 33 458–467. 10.1016/S0379-4172(06)60073-5 16722341

[B38] Wang K. Cui K. H. Liu G. L. Xie W. B. Yu H. H. Pan J. (2014). Identification of quantitative trait loci for phosphorus use efficiency traits in rice using a high density SNP map. *BMC Genet.* 15:155. 10.1186/s12863-014-0155-y 25551672 PMC4311488

[B39] Wang Y. Sun Y. J. Chen D. Y. Yu S. B. (2009). Analysis of quantitative trait loci in response to nitrogen and phosphorus deficiency in rice using chromosomal segment substitution lines. *Acta. Agron. Sin.* 35, 580–587. 10.1016/S1875-2780(08)60072-3

[B40] Wang Y. Zhang L. B. Nafisah A. Zhu L. H. Xu J. L. Li Z. K. (2013). Selection efficiencies for improving drought/salt tolerances and yield using introgression breeding in rice (Oryza sativa L.). *Crop J.* 1 134–142. 10.1016/j.cj.2013.07.006

[B41] Wei D. Cui K. H. Pan J. F. Ye G. Y. Xiang J. Nie L. (2011). Genetic dissection of grain nitrogen use efficiency and grain yield and their relationship in rice. *Field Crops Res.* 124 340–346. 10.1016/j.fcr.2011.07.003

[B42] Wei D. Cui K. H. Ye G. Y. Pan J. F. Xiang J. Huang J. (2012). QTL mapping for nitrogen-use efficiency and nitrogen deficiency tolerance traits in rice. *Plant Soil.* 359 281–295. 10.1007/s11104-012-1142-6

[B43] Wei J. L. Xu S. Z. (2016). A random model approach to QTL mapping in multi-parent advanced generation inter-cross (MAGIC) populations. *Genetics* 202 471–486. 26715662 10.1534/genetics.115.179945 PMC4788229

[B44] Yan J. B. Yang X. H. Shah T. Sánchez H. V. Li J. S. Warburton M. (2010). High-through put SNP genotyping with the golden gate assay in maize. *Mol. Breed.* 25 441–451. 10.1007/s11032-009-9343-2

[B45] Yoshida S. (1981). *Fundamentals of rice crop science.* Philippines: International Rice Research Institute, 135–147.

[B46] Yu J. M. Holland J. B. McMullen M. D. Buckler E. S. (2008). Genetic design and statistical power of nested association mapping in maize. *Genetics* 178 539–551. 10.1534/genetics.107.074245 18202393 PMC2206100

[B47] Zhang J. Liu Y. Zhang N. Hu B. Jin T. Xu H. (2019). NRT1.1B is associated with root microbiota composition and nitrogen use in field-grown rice. *Nat. Biotechnol.* 37 676–684. 31036930 10.1038/s41587-019-0104-4

[B48] Zhang Q. F. (2007). Strategies for developing green super rice. *Proc. Natl. Acad. Sci. USA.* 104 16402–16409. 10.1073/pnas.0708013104 17923667 PMC2034246

[B49] Zhao C. F. Zhou L. H. Zhang Y. D. Zhu Z. Chen T. Zhao Q. Y. (2014). QTL mapping for seedling traits associated with low-nitrogen tolerance using a set of advanced backcross introgression lines of rice. *Plant Breed.* 133 189–195. 10.1111/pbr.12123

[B50] Zhu Y. J. Chen K. Mi X. F. Chen T. X. Ali J. Ye G. Y. (2015). Identification and fine mapping of a stably expressed QTL for cold tolerance at the booting stage using an interconnected breeding psopulation in rice. *PLoS One* 10:e0145704. 10.1371/journal.pone.0145704 PMC4703131 26713764

